# Construction and validation of a predictive model of invasive adenocarcinoma in pure ground-glass nodules less than 2 cm in diameter

**DOI:** 10.1186/s12893-024-02341-2

**Published:** 2024-02-14

**Authors:** Mengchao Xue, Rongyang Li, Kun Wang, Wen Liu, Junjie Liu, Zhenyi Li, Guanqing Chen, Huiying Zhang, Hui Tian

**Affiliations:** Department of Thoracic Surgery, Qilu Hospital of Shandong University, Lixia District, Jinan, Shandong Province China

**Keywords:** Pulmonary pure glass nodule, Invasive adenocarcinoma, Prediction, Logical model, Nomogram

## Abstract

**Objectives:**

In this study, we aimed to develop a multiparameter prediction model to improve the diagnostic accuracy of invasive adenocarcinoma in pulmonary pure glass nodules.

**Method:**

We included patients with pulmonary pure glass nodules who underwent lung resection and had a clear pathology between January 2020 and January 2022 at the Qilu Hospital of Shandong University. We collected data on the clinical characteristics of the patients as well as their preoperative biomarker results and computed tomography features. Thereafter, we performed univariate and multivariate logistic regression analyses to identify independent risk factors, which were then used to develop a prediction model and nomogram. We then evaluated the recognition ability of the model via receiver operating characteristic (ROC) curve analysis and assessed its calibration ability using the Hosmer-Lemeshow test and calibration curves. Further, to assess the clinical utility of the nomogram, we performed decision curve analysis.

**Result:**

We included 563 patients, comprising 174 and 389 cases of invasive and non-invasive adenocarcinoma, respectively, and identified seven independent risk factors, namely, maximum tumor diameter, age, serum amyloid level, pleural effusion sign, bronchial sign, tumor location, and lobulation. The area under the ROC curve was 0.839 (95% CI: 0.798–0.879) for the training cohort and 0.782 (95% CI: 0.706–0.858) for the validation cohort, indicating a relatively high predictive accuracy for the nomogram. Calibration curves for the prediction model also showed good calibration for both cohorts, and decision curve analysis showed that the clinical prediction model has clinical utility.

**Conclusion:**

The novel nomogram thus constructed for identifying invasive adenocarcinoma in patients with isolated pulmonary pure glass nodules exhibited excellent discriminatory power, calibration capacity, and clinical utility.

**Supplementary Information:**

The online version contains supplementary material available at 10.1186/s12893-024-02341-2.

## Introduction

Owing to advances in diagnostic imaging and the widespread use of low-dose computerized tomography (LDCT) screening, an increasing number of pulmonary pure gross glass nodules (pGGNs) are being detected, causing alarm to patients [1–4]. A ground-glass nodule (GGN) is defined as a nodule with a slightly increased density that does not obscure underlying bronchial structures or vascular margins in high-resolution CT images [5]. Depending on the presence or absence of solid components, GGN can be classified as pure GGN (pGGN) and partially solid GGN. Notably, pGGNs are defined as GGNs without solid components [6].

Reportedly, the development of pGGNs progresses slowly, and at different pathological stages, they exhibit different growth patterns and show varying degrees of invasiveness. Some pGGN stages include: atypical adenomatous hyperplasia (AAH), adenocarcinoma in situ (AIS), microinvasive adenocarcinoma (MIA), and invasive adenocarcinoma (IAC) [7–10]. Currently, there are no uniform guidelines for GGN surgery. Some studies have shown that sublobar resection is acceptable for AAH, AIS, and MIA, but is unsuitable for IAC [11, 12]. With adequate surgical resection, patients with AIS and MIA exhibit approximately 100% disease-free survival [13–15], whereas the 5-year survival rate of patients with localized IAC have varies in the range70–90% [16, 17]. Therefore, the preoperative identification of the IAC is essential to help clinicians choose the correct procedure for their patients.

In clinical practice, identifying IAC manifesting as a pGGN is challenging. Attempts have been made in previous studies to distinguish between preinvasive adenocarcinomas and IACs presenting as pGGN [18–26]. Further, the correlation between pathological manifestations and nodules presenting as solid, partially solid, and pGGN have been investigated in other studies [27, 28]. However, in these previous studies, a wide variety of assessment factors were not identified. Additionally, the validity of some of these studies is limited by the small number of patients included.

In this study, we aimed to retrospectively analyze a relatively large number of patients with pGGN < 2 cm and develop a multiparametric predictive model and nomogram using patients’ clinical information, hematological findings, and imaging features to improve the ability of clinicians to diagnose IAC in pGGN and provide a basis for rational clinical decision-making.

## Materials and methods

This study was approved by the Ethics Committee of Qilu Hospital, Shandong University (registration number: KYLL-202008-023-1), and all the patients read and signed the informed consent form prior to this study, approving the use of their clinical information.

### Patient selection

**Fig. 1:**
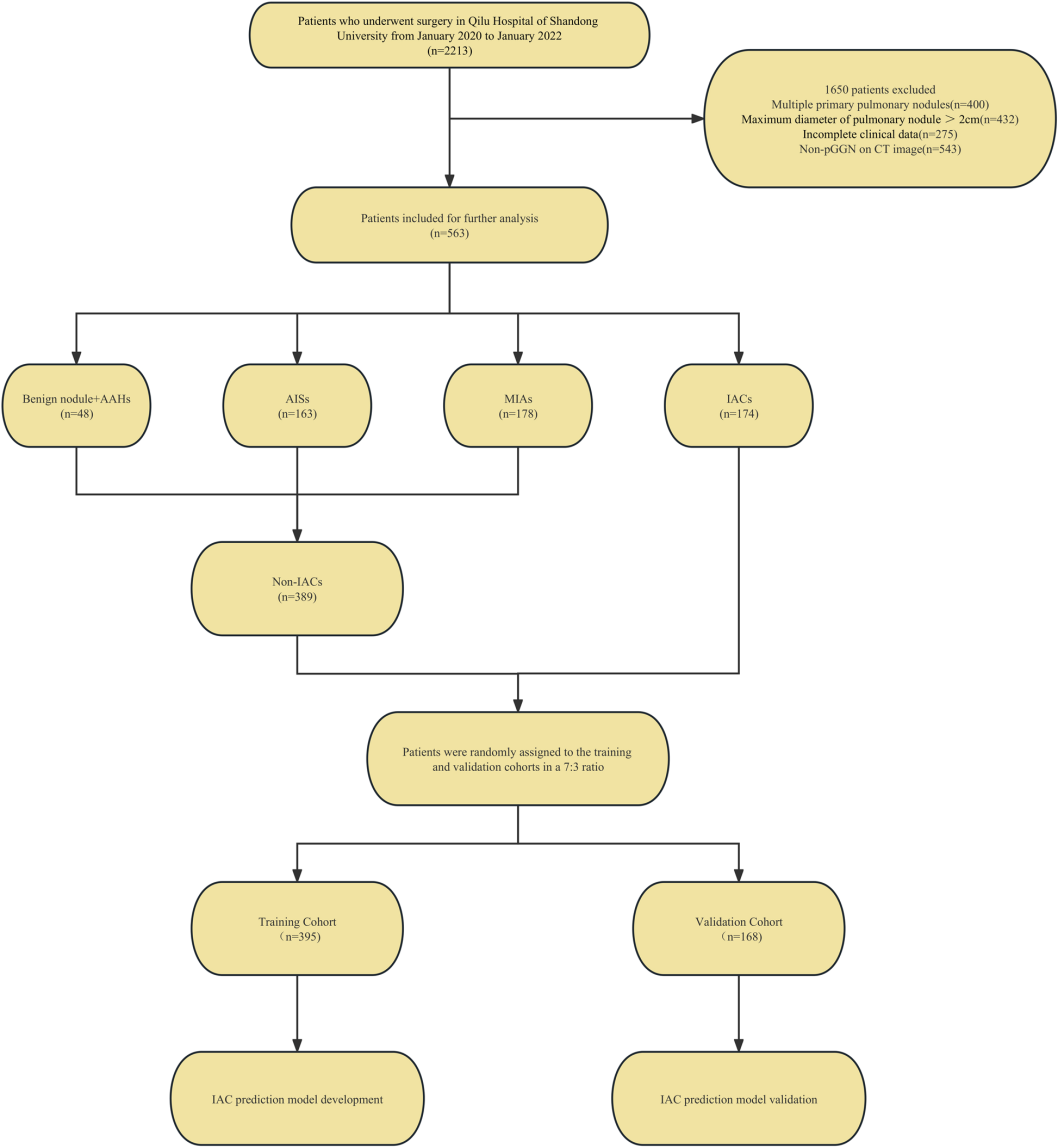
Flow diagram of patient selection through the study. AAH, atypical adenomatous hyperplasia; AIS, adenocarcinoma in situ; MIA, microinvasive adenocarcinoma; IAC, invasive adenocarcinoma

In this study, we retrospectively evaluated patients who underwent minimally invasive pneumonectomy with a clear pathology of pulmonary nodules at the Qilu Hospital of Shandong University between January 2020 and January 2022. The inclusion criteria were as follows: (1) patients with a single intrapulmonary nodule based on a chest CT scan performed within 1 month before surgery; (2) patients with lung nodules with maximum diameter ≤ 2 cm; (3) patients with pGGN without any solid component as indicated via CT imaging; (4) absence of pulmonary atelectasis and active inflammation based on lung images; (5) clear pathological findings obtained after surgical resection; (6) asymptomatic at diagnosis; and (7) no preoperative treatment. The exclusion criteria were as follows: (1) aged < 18 years, (2) history of thoracic surgery, (3) incomplete perioperative data, (4) history of malignant disease within the past 5 years, and (5) metastatic tumors. The patients included in our study were screened according to the inclusion and exclusion criteria (Fig. 1). Further, we used a random split-sample approach to randomly assign all the enrolled patients to either the training cohort or validation cohort at a ratio of 7:3. The training cohort was used to develop the prediction nomogram, while the validation cohort was used to verify the performance of the nomogram.

### Data collection and variable definitions

The following data were collected from the hospital database for all the eligible patients: (1) demographic data: sex, age, smoking history, body mass index (BMI), and preoperative comorbidities [hypertension, diabetes, and chronic obstructive pulmonary disease (COPD)]; (2) preoperative assessment data: American Society of Anesthesiologists (ASA) score, percentage of the predicted forced expiratory volume in one second (FEV1% predicted), and percentage of the predicted value of maximal voluntary ventilation (MVV% predicted); (3) laboratory blood test indicators: blood type, serum complement C1q, lactate dehydrogenase (LDH), serum amyloid (SA), serum 5’-nucleotidase (5’-NT), blood sugar, albumin, neutrophil, eosinophil, basophil, monocyte, lymphocyte, erythrocyte, hemoglobin, and platelet levels, derivative prognostic nutritional index (PNI), neutrophil-lymphocyte ratio (NLR), derived neutrophil-to-lymphocyte ratio (dNLR), platelet-lymphocyte ratio (PLR), monocyte-lymphocyte ratio (MLR), neutrophil-to-lymphocyte and platelet ratio (NLPR), systemic inflammatory response syndrome (SIRS), the aggregate index of systemic inflammation (AISI), systemic inflammation index (SII), and pan-immune-inflammation value (PIV); (4) lung cancer tumor markers: pro-gastrin-releasing peptide (pro-GRP), carcinoembryonic antigen (CEA), squamous cell carcinoma antigen (SCC), cytokeratin 19-fragment (cyfra21-1), carcinoma antigen 125 (CA125), and neuron-specific enolase (NSE) levels; (5) CT image characteristics: nodule location (central or peripheral), nodule shape (regular or irregular), spiculation (sunburst appearance), calcification, lobulation, cavitation signs, pleural adhesions, vascular penetration signs, bronchus signs, lymph node enlargement signs, pleural effusion signs, and maximum tumor diameter; and (6) postoperative pathological results. PNI, NLR, dNLR, MLR, NLPR, SIRI, AISI, SII, and PIV were calculated using the following expressions:

$$\mathrm{PNI}\;=\;\mathrm{serum}\;\mathrm{albumin}\;(\mathrm g/\mathrm L)\;+\;5\times\mathrm{total}\;\mathrm{lymphocyte}\;\mathrm{count}\;(\times109/\mathrm L)$$

$$\mathrm{NLR}=\mathrm{neutrophils}/\mathrm{lymphocytes};$$

$$\mathrm{PLR}=\mathrm{platelets}/\mathrm{lymphocytes};$$

$$\mathrm{dNRL}\;=\;\left[\mathrm{neutrophils}/\left(\mathrm{leukocytes}-\mathrm{neutrophils}\right)\right];$$

$$\mathrm{MLR}\;=\;\mathrm{monocytes}/\mathrm{lymphocytes};$$

$$\mathrm{NLPR}\;=\;\lbrack\mathrm{Neutrophils}/(\mathrm{lymphocytes}\;\times\;\mathrm{platelets})\rbrack;$$

$$\mathrm{SIRI}\;=\;\lbrack(\mathrm{neutrophils}\;\times\;\mathrm{monocytes})/\mathrm{lymphocytes})\rbrack;$$

$$\mathrm{AISI}\;=\;\lbrack(\mathrm{neutrophils}\;\times\;\mathrm{monocytes}\;\times\;\mathrm{platelets})/\mathrm{lymphocytes}\rbrack;$$

$$\mathrm{SII}\;=\;\lbrack(\mathrm{neutrophils}\;\times\;\mathrm{platelets})/\mathrm{lymphocytes})\rbrack;$$

$$\mathrm{PIV}\;=\;\lbrack(\mathrm{neutrophils}\;\times\;\mathrm{platelets}\;\times\;\mathrm{monocytes})/\mathrm{lymphocytes}\rbrack.$$

All chest CT examinations included the complete thorax and were performed in the supine position. Scans were obtained during deep inspiration and the patients holding their breath. The CT images were examined and interpreted by two radiologists with more than 5 years of experience in thoracic radiology. The two radiologists independently determined the characteristics of each image, while a third radiologist, with over 20 years of experience in thoracic radiology reassessed the measurements to check for discrepancies. Any discrepancies noted were resolved through discussion among the three radiologists. Central nodules were defined as those located in the bronchi, lobular bronchi, or segmental bronchi of the lungs, while peripheral nodules were defined as those located below the tertiary bronchi. Spiculation was defined as diffusion from the nodule edge into the lungs without contact with the pleural surface. Further, cavitation was defined as the presence of spaces filled with gas and considered regions of transparency or low attenuation. Pleural adhesion was defined as the linear attenuation of pleura or fissures from the nodule. Patterns in CT images, including delamination, central nodule, diffusion, or a popcorn pattern, were considered signs of calcification. A pulmonary artery passing through the nodule, as observed in the CT images was indicative of vascular penetration. Further, bronchial signs on the CT image showed direct bronchial engagement of the nodule. Lobulation was defined as a wavy or scalloped portion on the surface of a lesion, with strands stretching from the nodal edge to the lung parenchyma. Pleural effusion was defined based on a blunted angle of the rib diaphragm in the CT image. Lymph node enlargement was defined as a > 1-cm long lymph node axis in the CT image.

All pathological samples were fixed in formalin, stained with hematoxylin and eosin (H&E), and assessed by two experienced lung pathologists. The histopathological evaluation was performed by examining H&E-stained slides under a light microscope. All registered GGNs had clear pathological diagnoses. Pathological findings were divided into four groups: benign, AAH, AIS, MIA, and IAC. AAH, AIS, MIA, and IAC were based on the International Association for the Study of Lung Cancer (IASLC)/American Thoracic Society (ATS)/European Respiratory Society (ERS) classifications of lung in resected specimens [29]. Additionally, owing to the poor prognosis of patients with IAC, patients with benign lesions, AAH, AIS, and MIA were designated as the non-IAC group.

### Establishment of the predictive model

First, data for the training cohort were analyzed using univariate analysis to assess all the factors affecting IAC in pGGNs. Thereafter, all factors with *P* < 0.2 in the univariate analysis were included for further multivariate logistic regression analyses. A predictive model and nomogram were constructed using R statistical software (Windows version 4.2.1, http://www.r-project.org. The scores for each variable were calculated using a regression model, and the predicted probability of IAC in pGGNs was derived by summing the scores for each variable.

### Predictive model and nomogram performance

The performance of the predictive nomogram was evaluated based on its discriminatory power, calibration ability, and clinical utility. Discrimination describes the ability of a model to properly distinguish between incidents and non-incidents. Receiver operating characteristic (ROC) curves were used to evaluate the efficiency of the predictive nomograms [30]. Calibration gauges the extent to which predicted probabilities correspond to actual results. We used the Hosmer-Lemeshow test to assess the calibration capability of the model, and *P* > 0.05 was indicative of a satisfactory calibration ability [31]. Further evaluation of the calibration ability of the model was performed by constructing nomogram calibration curves. Internal verification was performed by repeating the bootstrap method 1,000 times [32]. Decision curve analysis (DCA) was used to assess the clinical utility of the predictive nomogram based on the net benefit of different threshold probabilities [33]. We determined the optimal cutoff value based on ROC curve analysis results for the training cohort when the Youden index (sensitivity + specificity − 1) reached its maximum value.

## Results

### Patient characteristics

The procedure for identifying and selecting eligible patients is illustrated in Fig. 1.

A total of 563 eligible patients were included in this study. Among these patients, there were 48 cases of benign nodules and AAH, 163 were AIS, 178 cases of MIA, and 174 cases of IAC. Further, we classified all the cases under the non-IACs (*n* = 389) or IACs (*n* = 174) groups, according to the criterion, “whether the nodule had IAC characteristics.” We then randomly assigned the enrolled patients to either the training cohort (*n* = 395) or validation cohort (*n* = 168) in a 7:3 ratio; no significant differences existed between the two cohorts with respect to any of the variable (Table 1). Thus, there were 273 non-IAC and 122 IAC cases in the training cohort and 116 non-IAC and 52 IAC cases in the validation cohort. The characteristics of the training and validation cohorts are presented in Table 2.

**Table 1:** Patients’ characteristics of the training cohort and validation cohort

Characteristics	All cohort (*N* = 563)	Validation cohort (*N* = 168)	Training cohort (*N* = 395)	*p*
IAC, n (%)				0.988
No	389 (69.1)	116 (69.0)	273 (69.1)	
Yes	174 (30.9)	52 (31.0)	122 (30.9)	
Gender, n (%)				0.785
Female	360 (63.9)	106 (63.1)	254 (64.3)	
Male	203 (36.1)	62 (36.9)	141 (35.7)	
Hypertension, n (%)				0.368
No	423 (75.1)	122 (72.6)	301 (76.2)	
Yes	140 (24.9)	46 (27.4)	94 (23.8)	
Diabetes, n (%)				0.221
No	503 (89.3)	146 (86.9)	357 (90.4)	
Yes	60 (10.7)	22 (13.1)	38 (9.6)	
COPD, n (%)				0.629
No	558 (99.1)	167 (99.4)	391 (99.0)	
Yes	5 (0.9)	1 (0.6)	4 (1.0)	
Smoking history, n (%)				0.78
Non-smoker	463 (82.2)	137 (81.5)	326 (82.5)	
Smoker	100 (17.8)	31 (18.5)	69 (17.5)	
Blood type, n (%)				0.435
A	166 (29.5)	47 (28.0)	119 (30.1)	
B	179 (31.8)	60 (35.7)	119 (30.1)	
AB	76 (13.5)	18 (10.7)	58 (14.7)	
O	142 (25.2)	43 (25.6)	99 (25.1)	
ASA, n (%)				0.239
1	77 (13.7)	17 (10.1)	60 (15.2)	
2	481 (85.4)	150 (89.3)	331 (83.8)	
3	5 (0.9)	1 (0.6)	4 (1.0)	
Location, n (%)				0.5
Centrality	47 (8.3)	12 (7.1)	35 (8.9)	
Peripherality	516 (91.7)	156 (92.9)	360 (91.1)	
Shape, n (%)				0.995
Regularity	335 (59.5)	100 (59.5)	235 (59.5)	
Irregularity	228 (40.5)	68 (40.5)	160 (40.5)	
Spiculation, n (%)				0.882
No	311 (55.2)	92 (54.8)	219 (55.4)	
Yes	252 (44.8)	76 (45.2)	176 (44.6)	
Cavitation sign, n (%)				0.599
No	500 (88.8)	151 (89.9)	349 (88.4)	
Yes	63 (11.2)	17 (10.1)	46 (11.6)	
Calcification, n (%)				0.356
No	561 (99.6)	168 (100.0)	393 (99.5)	
Yes	2 (0.4)	0 (0.0)	2 (0.5)	
Vascular penetration sign, n (%)				0.234
No	207 (36.8)	68 (40.5)	139 (35.2)	
Yes	356 (63.2)	100 (59.5)	256 (64.8)	
Pleural adhesions, n (%)				0.688
No	365 (64.8)	111 (66.1)	254 (64.3)	
Yes	198 (35.2)	57 (33.9)	141 (35.7)	
Bronchus sign, n (%)				0.27
No	458 (81.3)	132 (78.6)	326 (82.5)	
Yes	105 (18.7)	36 (21.4)	69 (17.5)	
Lobulation, n (%)				0.7
No	448 (79.6)	132 (78.6)	316 (80.0)	
Yes	115 (20.4)	36 (21.4)	79 (20.0)	
Lymph node enlargement sign, n (%)				0.422
No	508 (90.2)	149 (88.7)	359 (90.9)	
Yes	55 (9.8)	19 (11.3)	36 (9.1)	
Pleural effusion sign, n (%)				0.356
No	561 (99.6)	168 (100.0)	393 (99.5)	
Yes	2 (0.4)	0 (0.0)	2 (0.5)	
Albumin (g/L), median (IQR)	60.10 (58.00, 62.05)	60.10 (57.98, 62.30)	60.10 (58.00, 61.90)	0.825
Lymphocyte (×109/L), median (IQR)	1.84 (1.49, 2.23)	1.81 (1.48, 2.19)	1.86 (1.50, 2.26)	0.517
PNI (%), median (IQR)	69.35 (66.93, 71.90)	69.45 (66.99, 71.48)	69.30 (66.85, 72.03)	0.958
Neutrophil (×109/L), median (IQR)	2.97 (2.43, 3.66)	2.95 (2.45, 3.90)	2.97 (2.43, 3.50)	0.375
Eosinophil (×109/L), median (IQR)	0.10 (0.06, 0.16)	0.10 (0.07, 0.16)	0.09 (0.06, 0.16)	0.573
Basophil (×109/L), median (IQR)	0.03 (0.02, 0.04)	0.03 (0.02, 0.04)	0.03 (0.02, 0.04)	0.508
Monocyte (×109/L), median (IQR)	0.40 (0.33, 0.49)	0.42 (0.35, 0.52)	0.40 (0.33, 0.49)	0.089
Erythrocyte (×1012/L), median (IQR)	4.48 (4.20, 4.83)	4.48 (4.16, 4.81)	4.48 (4.23, 4.84)	0.722
Hemoglobin (g/L), median (IQR)	136.00 (128.00, 146.50)	135.50 (127.00, 144.25)	136.00 (129.00, 147.00)	0.351
Platelet (×109/L), median (IQR)	236.00 (206.00, 272.50)	237.00 (202.75, 272.50)	235.00 (207.50, 272.50)	0.991
NLR (%), median (IQR)	1.64 (1.27, 2.09)	1.70 (1.25, 2.15)	1.62 (1.28, 2.06)	0.423
PLR (%), median (IQR)	130.00 (105.50, 158.60)	133.50 (104.57, 160.84)	129.21 (106.15, 156.53)	0.531
MLR (%), median (IQR)	0.22 (0.18, 0.27)	0.22 (0.18, 0.28)	0.21 (0.17, 0.27)	0.149
dNLR (%), median (IQR)	1.25 (1.00, 1.54)	1.26 (0.96, 1.57)	1.25 (1.00, 1.53)	0.753
NLPR (%), median (IQR)	0.01 (0.01, 0.01)	0.01 (0.01, 0.01)	0.01 (0.01, 0.01)	0.6
SIRI (%), median (IQR)	0.63 (0.47, 0.92)	0.65 (0.47, 1.03)	0.62 (0.47, 0.90)	0.153
AISI (%), median (IQR)	147.97 (103.82, 226.98)	156.63 (104.56, 253.41)	146.32 (103.60, 220.58)	0.155
SII (%), median (IQR)	379.78 (288.68, 505.98)	387.88 (281.58, 537.30)	375.44 (290.75, 488.74)	0.399
PIV (%), median (IQR)	147.97 (103.82, 226.98)	156.63 (104.56, 253.41)	146.32 (103.60, 220.58)	0.155
Blood sugar(mmol/L), median (IQR)	5.09 (4.70, 5.58)	5.11 (4.71, 5.62)	5.07 (4.70, 5.58)	0.591
Complement C1q(mg/L), median (IQR)	171.00 (153.05, 190.85)	167.00 (151.10, 190.50)	171.60 (153.85, 190.85)	0.341
LDH (U/L), median (IQR)	191.00 (169.50, 215.50)	195.00 (166.00, 217.00)	191.00 (171.00, 215.00)	0.991
SA (mg/dL), median (IQR)	53.10 (48.90, 57.30)	52.70 (48.80, 57.90)	53.20 (48.95, 57.20)	0.928
5’-NT (U/L), median (IQR)	4.00 (3.00, 5.00)	4.00 (3.00, 5.00)	4.00 (3.00, 5.00)	0.801
Pro-GRP (pg/mL), median (IQR)	41.96 (33.02, 45.84)	41.96 (32.49, 45.51)	41.96 (33.05, 46.17)	0.483
SCC (ng/mL), median (IQR)	1.05 (0.80, 1.70)	1.07 (0.73, 1.83)	1.03 (0.80, 1.69)	0.735
Cyfra21-1 (ng/mL), median (IQR)	2.28 (1.62, 2.54)	2.20 (1.60, 2.43)	2.31 (1.64, 2.56)	0.57
CEA (ng/mL), median (IQR)	2.30 (1.41, 2.60)	2.26 (1.39, 2.54)	2.32 (1.42, 2.61)	0.613
CA125 (U/mL), median (IQR)	10.72 (7.50, 11.55)	10.70 (7.40, 11.60)	10.72 (7.53, 11.35)	0.663
NSE (ng/mL), median (IQR)	19.45 (15.85, 21.00)	19.30 (15.57, 20.33)	19.45 (16.30, 21.05)	0.359
Age (years), median (IQR)	56.00 (48.00, 63.00)	57.00 (49.00, 63.00)	56.00 (48.00, 63.00)	0.464
BMI (kg/m2), median (IQR)	24.57 (22.48, 26.43)	24.76 (22.58, 26.46)	24.45 (22.42, 26.34)	0.391
FEV1% predicted (%), median (IQR)	105.97 (95.56, 114.79)	104.89 (94.26, 113.56)	106.05 (96.50, 115.71)	0.129
MVV% predicted (%), median (IQR)	104.79 (91.40, 116.59)	103.80 (90.24, 113.51)	105.00 (91.90, 118.06)	0.288
Maximum diameter (cm), median (IQR)	1.00 (0.70, 1.40)	1.00 (0.80, 1.30)	1.00 (0.70, 1.40)	0.383

*IAC *Invasive adenocarcinomam, *COPD *Chronic obstructive pulmonary diseases, *ASA *American Society of Anesthesiologists, *PNI *Prognostic nutritional index, *NLR *Neutrophil-lymphocyte ratio, *PLR *Platelet-lymphocyte ratio, *MLR *Monocyte-lymphocyte ratio, *dNLR *Derived neutrophil-to-lymphocyte ratio, *NLPR *Neutrophil to lymphocyte and platelet ratio, *SIRI *Systemic inflammatory response syndrome, *AISI *Aggregate index of systemic inflammation, *SII *Systemic inflammation index, *PIV *Pan-immune-inflammation value, *LDH *Lactate dehydrogenase, *SA *Serum amyloid, *5'-NT *5'-nucleotidase, *Pro-GRP *Pro-gastrin-releasing peptide, *SCC *Squamous cell carcinoma, * Cyfra21-1 *Cytokeratin 19-fragments, *CEA *Carcinoembryonic antigen, *CA125 *Carcinoma antigen 125, *NSE *Neuron-specific enolase, *BMI *Body mass index, *FEV1 *Forced expiratory volume in one second, *MVV *Maximal voluntary ventilation

*P*-value for the comparison between training cohort and validation cohort

**Table 2:** Clinical characteristics of patients with IACs and Non-IACs in the training and validation cohorts

Characteristics	Training Cohort(*n* = 395)			Validation cohort(*n* = 168)		
	Non-IACs(*n* = 273)	IACs(*n* = 122)	*P*	Non-IACs(*n* = 116)	IACs(*n* = 52)	*P*
Gender, n (%)			0.009			0.779
Female	187 (68.5)	67 (54.9)		74 (63.8)	32 (61.5)	
Male	86 (31.5)	55 (45.1)		42 (36.2)	20 (38.5)	
Hypertension, n (%)			0.075			0.643
No	215 (78.8)	86 (70.5)		83 (71.6)	39 (75.0)	
Yes	58 (21.2)	36 (29.5)		33 (28.4)	13 (25.0)	
Diabetes, n (%)			0.786			0.114
No	246 (90.1)	111 (91.0)		104 (89.7)	42 (80.8)	
Yes	27 (9.9)	11 (9.0)		12 (10.3)	10 (19.2)	
COPD, n (%)			0.406			0.134
No	271 (99.3)	120 (98.4)		116 (100.0)	51 (98.1)	
Yes	2 (0.7)	2 (1.6)		0 (0.0)	1 (1.9)	
Smoking history, n (%)			0.013			0.798
Non-smoker	234 (85.7)	92 (75.4)		94 (81.0)	43 (82.7)	
Smoker	39 (14.3)	30 (24.6)		22 (19.0)	9 (17.3)	
Blood type, n (%)			0.032			0.92
A	88 (32.2)	31 (25.4)		31 (26.7)	16 (30.8)	
B	74 (27.1)	45 (36.9)		41 (35.3)	19 (36.5)	
AB	35 (12.8)	23 (18.9)		13 (11.2)	5 (9.6)	
O	76 (27.8)	23 (18.9)		31 (26.7)	12 (23.1)	
ASA, n (%)			0.541			0.262
1	44 (16.1)	16 (13.1)		13 (11.2)	4 (7.7)	
2	227 (83.2)	104 (85.2)		103 (88.8)	47 (90.4)	
3	2 (0.7)	2 (1.6)		0 (0.0)	1 (1.9)	
Location, n (%)			< 0.001			0.853
Centrality	15 (5.5)	20 (16.4)		8 (6.9)	4 (7.7)	
Peripherality	258 (94.5)	102 (83.6)		108 (93.1)	48 (92.3)	
Shape, n (%)			< 0.001			0.007
Regularity	189 (69.2)	46 (37.7)		77 (66.4)	23 (44.2)	
Irregularity	84 (30.8)	76 (62.3)		39 (33.6)	29 (55.8)	
Spiculation, n (%)			< 0.001			0.03
No	171 (62.6)	48 (39.3)		70 (60.3)	22 (42.3)	
Yes	102 (37.4)	74 (60.7)		46 (39.7)	30 (57.7)	
Cavitation sign, n (%)			0.543			0.336
No	243 (89.0)	106 (86.9)		106 (91.4)	45 (86.5)	
Yes	30 (11.0)	16 (13.1)		10 (8.6)	7 (13.5)	
Calcification, n (%)			0.034			NA
No	273 (100.0)	120 (98.4)		116 (100.0)	52 (100.0)	
Yes	0 (0.0)	2 (1.6)		0(0)	0(0)	
Vascular penetration sign, n (%)			< 0.001			0.04
No	112 (41.0)	27 (22.1)		53 (45.7)	15 (28.8)	
Yes	161 (59.0)	95 (77.9)		63 (54.3)	37 (71.2)	
Pleural adhesions, n (%)			< 0.001			0.059
No	195 (71.4)	59 (48.4)		82 (70.7)	29 (55.8)	
Yes	78 (28.6)	63 (51.6)		34 (29.3)	23 (44.2)	
Bronchus sign, n (%)			< 0.001			0.005
No	245 (89.7)	81 (66.4)		98 (84.5)	34 (65.4)	
Yes	28 (10.3)	41 (33.6)		18 (15.5)	18 (34.6)	
Lobulation, n (%)			< 0.001			0.001
No	236 (86.4)	80 (65.6)		99 (85.3)	33 (63.5)	
Yes	37 (13.6)	42 (34.4)		17 (14.7)	19 (36.5)	
Lymph node enlargement sign, n (%)			0.142			0.555
No	252 (92.3)	107 (87.7)		104 (89.7)	45 (86.5)	
Yes	21 (7.7)	15 (12.3)		12 (10.3)	7 (13.5)	
Pleural effusion sign, n (%)			0.558			NA
No	272 (99.6)	121 (99.2)		116 (100.0)	52 (100.0)	
Yes	1 (0.4)	1 (0.8)		0(0)	0(0)	
Albumin (g/L), median (IQR)	60.00 (57.90, 61.90)	60.45 (58.42, 61.98)	0.188	59.85 (57.58, 61.92)	60.60 (58.58, 63.02)	0.032
Lymphocyte (×109/L), median (IQR)	1.86 (1.53, 2.19)	1.86 (1.45, 2.29)	0.932	1.81 (1.47, 2.19)	1.83 (1.49, 2.19)	0.986
PNI (%), median (IQR)	69.20 (66.90, 71.90)	69.68 (66.46, 72.35)	0.278	69.47 (66.58, 71.18)	69.40 (67.79, 72.03)	0.21
Neutrophil (×109/L), median (IQR)	2.96 (2.42, 3.46)	3.00 (2.48, 3.80)	0.323	3.03 (2.55, 3.91)	2.87 (2.38, 3.86)	0.595
Eosinophil (×109/L), median (IQR)	0.09 (0.06, 0.15)	0.12 (0.06, 0.21)	0.033	0.10 (0.07, 0.16)	0.09 (0.06, 0.16)	0.603
Basophil (×109/L), median (IQR)	0.03 (0.02, 0.04)	0.03 (0.02, 0.04)	0.284	0.03 (0.02, 0.04)	0.03 (0.02, 0.03)	0.184
Monocyte (×109/L), median (IQR)	0.39 (0.32, 0.48)	0.42 (0.33, 0.49)	0.117	0.42 (0.35, 0.51)	0.42 (0.33, 0.54)	0.739
Erythrocyte (×1012/L), median (IQR)	4.46 (4.18, 4.75)	4.54 (4.29, 4.93)	0.059	4.44 (4.16, 4.78)	4.55 (4.17, 4.86)	0.291
Hemoglobin (g/L), median (IQR)	135.00 (128.00, 144.00)	139.50 (130.00, 149.00)	0.041	134.00 (127.00, 143.25)	139.00 (126.75, 148.50)	0.33
Platelet (×109/L), median (IQR)	232.00 (207.00, 272.00)	239.00 (209.50, 276.00)	0.622	237.50 (201.25, 281.50)	234.50 (205.25, 257.25)	0.616
NLR (%), median (IQR)	1.60 (1.28, 2.03)	1.67 (1.27, 2.23)	0.363	1.71 (1.35, 2.11)	1.67 (1.22, 2.19)	0.826
PLR (%), median (IQR)	128.66 (107.41, 153.85)	131.50 (104.25, 166.56)	0.587	137.07 (106.19, 162.07)	130.97 (99.35, 159.77)	0.746
MLR (%), median (IQR)	0.21 (0.17, 0.27)	0.22 (0.18, 0.28)	0.161	0.22 (0.18, 0.27)	0.22 (0.19, 0.28)	0.918
dNLR (%), median (IQR)	1.25 (1.00, 1.50)	1.26 (1.01, 1.63)	0.536	1.26 (1.00, 1.54)	1.26 (0.91, 1.60)	0.802
NLPR (%), median (IQR)	0.01 (0.01, 0.01)	0.01 (0.00, 0.01)	0.496	0.01 (0.01, 0.01)	0.01 (0.01, 0.01)	0.862
SIRI (%), median (IQR)	0.59 (0.46, 0.89)	0.66 (0.48, 0.92)	0.132	0.65 (0.49, 0.95)	0.63 (0.45, 1.14)	0.809
AISI (%), median (IQR)	143.50 (96.94, 213.11)	161.29 (112.10, 227.89)	0.119	161.00 (109.14, 259.27)	152.14 (95.38, 228.30)	0.869
SII (%), median (IQR)	362.80 (284.64, 487.08)	392.56 (307.21, 513.13)	0.208	393.73 (282.70, 541.46)	369.95 (277.57, 516.84)	0.588
PIV (%), median (IQR)	143.50 (96.94, 213.11)	161.29 (112.10, 227.89)	0.119	161.00 (109.14, 259.27)	152.14 (95.38, 228.30)	0.869
Blood sugar(mmol/L), median (IQR)	5.04 (4.69, 5.52)	5.10 (4.71, 5.67)	0.455	5.04 (4.69, 5.61)	5.22 (4.89, 5.71)	0.092
Complement C1q(mg/L), median (IQR)	171.60 (153.90, 190.10)	171.80 (153.98, 192.02)	0.811	169.50 (149.85, 190.50)	166.70 (154.23, 188.52)	0.76
LDH (U/L), median (IQR)	191.00 (172.00, 212.00)	188.50 (167.25, 218.75)	0.845	195.00 (167.50, 214.50)	193.50 (161.50, 219.25)	0.825
SA (mg/dL), median (IQR)	52.80 (48.80, 56.50)	53.40 (49.02, 58.27)	0.207	52.50 (48.77, 57.28)	53.45 (49.53, 59.20)	0.434
5’-NT (U/L), median (IQR)	4.00 (3.00, 5.00)	4.00 (3.00, 5.00)	0.653	4.00 (3.00, 5.00)	4.00 (3.00, 5.00)	0.251
Pro-GRP (pg/mL), median (IQR)	41.96 (33.49, 46.73)	41.83 (32.18, 45.64)	0.479	41.96 (32.49, 45.72)	41.64 (32.70, 42.81)	0.724
SCC (ng/mL), median (IQR)	1.03 (0.82, 1.73)	1.04 (0.72, 1.59)	0.258	1.03 (0.76, 1.76)	1.09 (0.70, 1.83)	0.684
Cyfra21-1 (ng/mL), median (IQR)	2.28 (1.55, 2.37)	2.32 (1.82, 2.73)	0.035	2.19 (1.50, 2.44)	2.27 (1.67, 2.43)	0.563
CEA (ng/mL), median (IQR)	2.28 (1.36, 2.43)	2.32 (1.65, 3.14)	0.013	2.04 (1.26, 2.45)	2.32 (1.91, 2.64)	0.052
CA125 (U/mL), median (IQR)	10.72 (7.52, 11.20)	10.72 (7.64, 12.28)	0.366	10.72 (7.40, 11.72)	10.22 (7.65, 10.72)	0.613
NSE (ng/mL), median (IQR)	19.45 (16.70, 20.50)	19.35 (14.90, 22.05)	0.505	19.42 (15.75, 21.00)	17.60 (15.40, 19.49)	0.3
Age (years), median (IQR)	55.00 (46.00, 60.00)	61.00 (52.00, 67.00)	< 0.001	56.00 (47.75, 62.00)	60.00 (53.50, 65.25)	0.041
BMI (kg/m2), median (IQR)	24.39 (22.27, 26.35)	24.57 (22.62, 26.20)	0.712	24.65 (22.48, 26.32)	24.90 (22.65, 27.14)	0.448
FEV1% predicted (%), median (IQR)	105.92 (96.93, 113.73)	109.20 (95.46, 118.07)	0.204	105.32 (94.40, 113.67)	102.44 (93.88, 112.58)	0.747
MVV% predicted (%), median (IQR)	103.87 (91.91, 116.64)	106.58 (91.88, 119.90)	0.183	103.80 (91.20, 115.39)	103.85 (88.55, 112.29)	0.717
Maximum diameter (cm), median (IQR)	0.80 (0.70, 1.10)	1.40 (1.20, 1.70)	< 0.001	0.80 (0.70, 1.00)	1.20 (1.00, 1.50)	< 0.001

*IAC *Invasive adenocarcinoma, *COPD *Chronic obstructive pulmonary diseases, *ASA *American Society of Anesthesiologists, *PNI *Prognostic nutritional index, *NLR *Neutrophil-lymphocyte ratio,  *PLR *Platelet-lymphocyte ratio, *MLR *Monocyte-lymphocyte ratio, *dNLR *derived neutrophil-to-lymphocyte ratio, *NLPR *Neutrophil to lymphocyte and platelet ratio, *SIRI *Systemic inflammatory response syndrome, *AISI *Aggregate index of systemic inflammation, *SII *Systemic inflammation index, *PIV *Pan-immune-inflammation value, *LDH *Lactate dehydrogenase, *SA *Serum amyloid, * 5'-NT *5'-nucleotidase, *Pro-GRP *Pro-gastrin-releasing peptide, *SCC *Squamous cell carcinoma, *Cyfra21-1 *Cytokeratin 19-fragments, *CEA *Carcinoembryonic antigen, *CA125 *Carcinoma antigen 125, *NSE *Neuron-specific enolase, *BMI *Body mass index, *FEV1 *Forced expiratory volume in one second, *MVV *Maximal voluntary ventilation

### Identification of risk factors for pGGNs measuring ≤ 2 cm

**Fig. 2:**
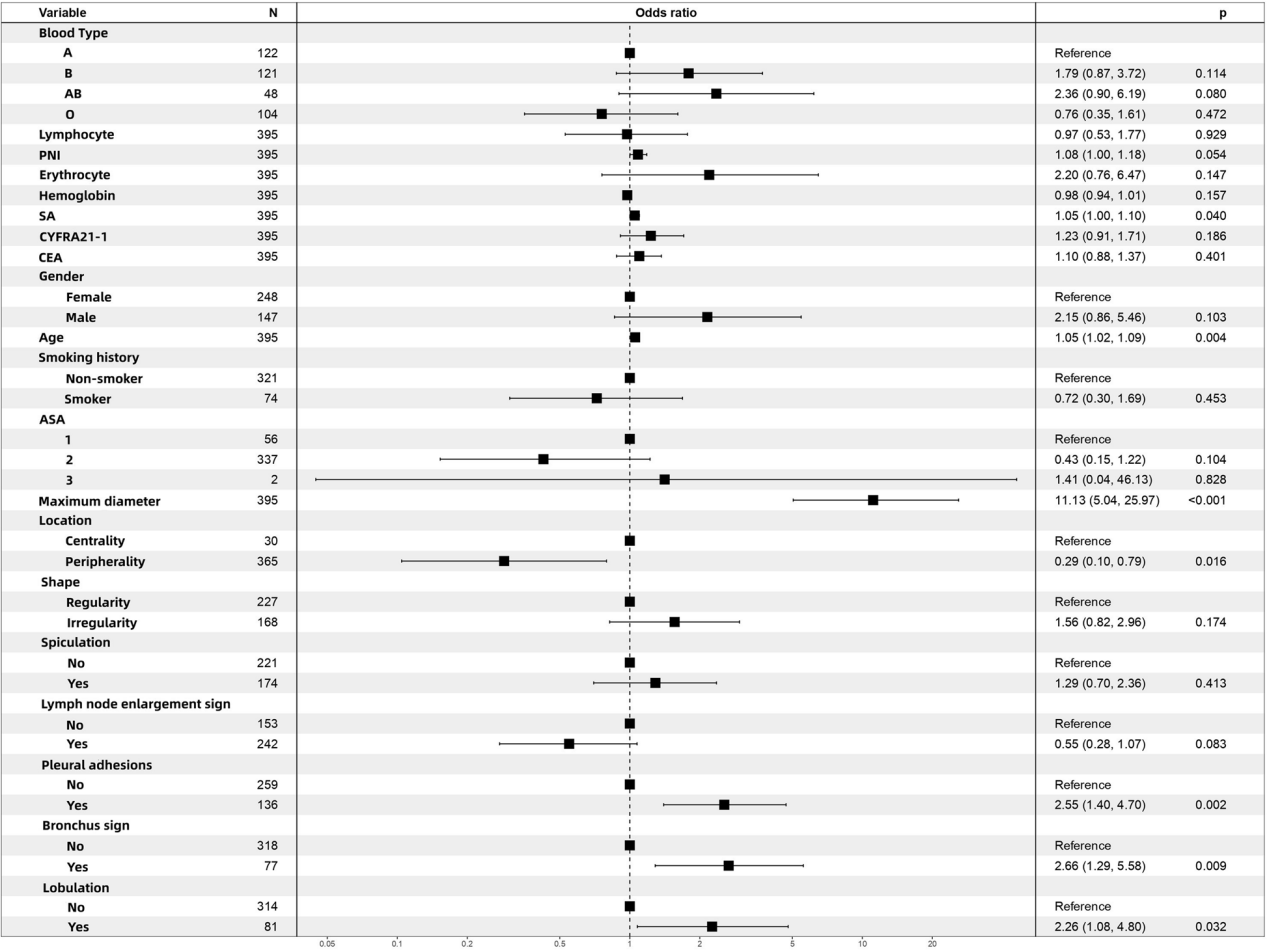
Multi-factor logistic regression analysis of forest plots. PNI, prognostic nutritional index; SA, serum amyloid; Cyfra21-1, cytokeratin 19-fragments; CEA, carcinoembryonic antigen; ASA, American Society of Anesthesiologists

Univariate and multivariate logistic regression analyses were performed on the training cohort to explore the independent risk factors for IACs in pGGNs. Table 3 shows the results of the logistic regression analysis. Via univariate analysis, up to 20 factors were identified as potential risk factors for IACs in pGGNs measuring ***≤*** 2 cm (*P* < 0.2). Further multivariate logistic regression analysis based on these 20 univariate variables with *P* < 0.2 led to the identification of seven indicators, namely, maximum tumor diameter [odds ratio (OR) = 11.130; 95% confidence interval (CI): 5.044–25.966; *P* < 0.001]; age (OR = 1.054; 95% CI: 1.018–1.094; *P* = 0.004); SA (OR = 1.050; 95% CI:1.002–1.100; *P* = 0.04); pleural effusion sign (yes vs. no; OR = 2.548; 95% CI: 1.398–4.700; *P* = 0.002); bronchus sign (yes vs. no; OR = 2.662; 95% CI: 1.286–5.576; *P* = 0.009); tumor location (centrality vs. peripherality; OR = 0.288; 95% CI: 0.104–0.794; *P* = 0.016); and lobulation (yes vs. no; OR = 2.260; 95% CI: 1.078–4.799; *P* = 0.032). The forest plot for the multivariate logistic regression analysis is shown in Fig. 2.

**Table 3:** Univariate and multivariate logistic regression analysis of IAC factors of pGGNs within 2 cm in a training cohort

Variables	Univariate analysis		Multivariate analysis	
	OR (95%CI)	*P* value	OR (95%CI)	*P* value
Gender				
Female	Ref.	Ref.	Ref.	Ref.
Male	1.615 (1.043, 2.499)	0.031	2.154 (0.859, 5.458)	0.103
Smoking history				
Non-smoker	Ref.	Ref.	Ref.	Ref.
Smoker	1.697 (0.999, 2.856)	0.048	0.721 (0.304, 1.685)	0.453
Pleural effusion sign				
No	Ref.	Ref.	Ref.	Ref.
Yes	2.779 (1.785, 4.344)	< 0.001	2.548 (1.398, 4.700)	0.002
Bronchus sign				
No	Ref.	Ref.	Ref.	Ref.
Yes	4.103 (2.452, 6.935)	< 0.001	2.662 (1.286, 5.576)	0.009
Location				
Centrality	Ref.	Ref.	Ref.	Ref.
Peripherality	0.415 (0.194, 0.884)	0.022	0.288 (0.104, 0.794)	0.016
Lobulation				
No	Ref.	Ref.	Ref.	Ref.
Yes	2.760 (1.669, 4.575)	< 0.001	2.260 (1.078, 4.799)	0.032
Vascular penetration sign				
No	Ref.	Ref.	Ref.	Ref.
Yes	2.372 (1.492, 3.846)	< 0.001	0.549 (0.275, 1.074)	0.083
Shape				
Regularity	Ref.	Ref.	Ref.	Ref.
Irregularity	3.798 (2.436, 5.993)	< 0.001	1.559 (0.819, 2.962)	0.174
Spiculation				
No	Ref.	Ref.	Ref.	Ref.
Yes	3.105 (2.001, 4.865)	< 0.001	1.288 (0.699, 2.360)	0.413
Blood type				
A	Ref.	Ref.	Ref.	Ref.
B	1.550 (0.898, 2.698)	0.117	1.790 (0.875, 3.722)	0.114
AB	2.009 (0.991, 4.055)	0.051	2.356 (0.898, 6.187)	0.08
O	0.986 (0.541, 1.788)	0.964	0.757 (0.352, 1.609)	0.472
ASA				
1	Ref.	Ref.	Ref.	Ref.
2	1.982 (1.020, 4.169)	0.054	0.425 (0.153, 1.222)	0.104
3	4.091 (0.154, 109.087)	0.332	1.412 (0.045, 46.131)	0.828
Maximum diameter	16.916 (8.943, 33.624)	< 0.001	11.130 (5.044, 25.966)	< 0.001
Age	1.056 (1.033, 1.081)	< 0.001	1.054 (1.018, 1.094)	0.004
SA	1.046 (1.013, 1.081)	0.006	1.050 (1.002, 1.100)	0.04
PNI	1.063 (1.013, 1.120)	0.016	1.084 (1.002, 1.183)	0.054
Erythrocyte	1.801 (1.130, 2.895)	0.014	2.196 (0.759, 6.471)	0.147
Hemoglobin	1.015 (1.000, 1.030)	0.048	0.975 (0.941, 1.010)	0.157
Cyfra21_1	1.438 (1.137, 1.846)	0.003	1.232 (0.911, 1.705)	0.186
CEA	1.198 (1.017, 1.421)	0.032	1.099 (0.877, 1.366)	0.401
Lymphocyte	1.445 (1.012, 2.068)	0.043	0.973 (0.527, 1.769)	0.929
Hypertension				
No	Ref.	Ref.		
Yes	1.372 (0.836, 2.227)	0.205		
Diabetes				
No	Ref.	Ref.		
Yes	0.966 (0.471, 1.887)	0.922		
COPD				
NO	Ref.	Ref.		
Yes	2.258 (0.268, 19.002)	0.418		
Calcification				
No	Ref.	Ref.		
Yes	4818859.448 (0.000, NA)	0.98		
Cavitation sign				
No	Ref.	Ref.		
Yes	1.181 (0.581, 2.307)	0.633		
Pleural effusion sign				
No	Ref.	Ref.		
Yes	0	0.981		
Lymph node enlargement sign				
No	Ref.	Ref.		
Yes	1.479 (0.715, 2.972)	0.278		
AISI	1.000 (0.999, 1.001)	0.811		
Albumin	1.037 (0.983, 1.099)	0.204		
Basophil	4.812 (0.191, 336.295)	0.339		
Blood sugar	1.064 (0.905, 1.248)	0.432		
BMI	1.014 (0.947, 1.086)	0.681		
CA125	1.010 (0.978, 1.042)	0.521		
Complement C1q	1.000 (0.993, 1.007)	0.996		
dNLR	0.845 (0.536, 1.309)	0.46		
Eosinophil	1.588 (0.639, 4.419)	0.312		
FEV1% predicted (%)	1.005 (0.992, 1.019)	0.438		
IDH	1.000 (0.993, 1.006)	0.917		
MLR	1.054 (0.558, 1.769)	0.83		
Monocyte	1.205 (0.879, 1.952)	0.279		
MVV% predicted (%)	1.001 (0.998, 1.005)	0.396		
Neutrophil	1.077 (0.877, 1.317)	0.473		
NLPR	0.003	0.813		
NLR	0.923 (0.685, 1.226)	0.589		
NSE	1.010 (0.975, 1.045)	0.572		
PIV	1.000 (0.999, 1.001)	0.811		
Platelet	1.000 (0.997, 1.004)	0.81		
PLR	0.998 (0.994, 1.002)	0.41		
Pro-GRP	0.997 (0.980, 1.011)	0.663		
SCC	0.849 (0.583, 1.167)	0.361		
SII	1.000 (0.999, 1.001)	0.465		
SIRI	1.028 (0.836, 1.236)	0.748		
5’-NT	1.004 (0.886, 1.121)	0.944		

*IAC *Invasive adenocarcinoma, *COPD *Chronic obstructive pulmonary diseases, *ASA *American Society of Anesthesiologists, *PNI *Prognostic nutritional index, *NLR *Neutrophil-lymphocyte ratio, *PLR *Platelet-lymphocyte ratio, *MLR *Monocyte-lymphocyte ratio, *dNLR *derived neutrophil-to-lymphocyte ratio, *NLPR *Neutrophil to lymphocyte and platelet ratio, *SIRI *Systemic inflammatory response syndrome, *AISI *Aggregate index of systemic inflammation, *SII *Systemic inflammation index, *PIV *Pan-immune-inflammation value, *LDH*, Lactate dehydrogenase, *SA *Serum amyloid, *5'-NT *5'-nucleotidase, *Pro-GRP *Pro-gastrin-releasing peptide, *SCC *Squamous cell carcinoma, *Cyfra21-1 *Cytokeratin 19-fragments, *CEA *Carcinoembryonic antigen, *CA125 *Carcinoma antigen 125, *NSE *Neuron-specific enolase, *BMI *Body mass index, *FEV1 *Forced expiratory volume in one second, *MVV *Maximal voluntary ventilation

### Nomogram establishment

**Fig. 3:**
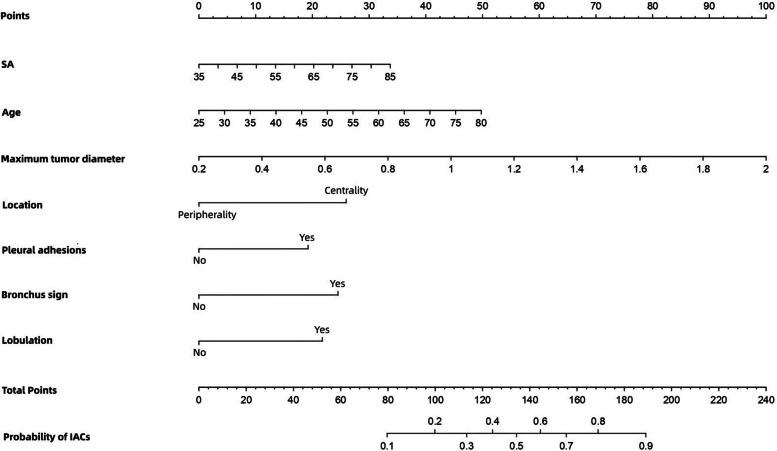
Nomogram for predicting the probability of IAC for pGGN ≤ 2 cm. SA, serum amyloid

All seven independent risk factors for pGGNs measuring ≤ 2 cm were included in the logistic regression models. Details regarding the prediction model are presented in Table 4. The probability of the occurrence of IACs in small pGGNs was then calculated according to the following equation: ln (p/1-p) = 2.41 × maximum tumor diameter + 0.053 × age + 0.049 × SA – 1.245 × tumor location (centrality = 0; peripherality = 1) + 0.935 × pleural adhesions (no = 0; yes = 1) + 0.979 × bronchus sign (no = 0; yes = 1) + 0.815 × lobulation (no = 0; yes = 1) – 12.759. Further, we plotted the predicted nomogram for the probability of IACs in pGGNs of size ≤ 2 cm using R statistical software, based on the above equation (Fig. 3). The nomogram comprised 10 axes, with axes 2–8 representing the seven variables in the prediction model. By drawing a line vertically to the highest-point axis, the estimated score of each risk factor could be computed and added to obtain the total risk score, which was then used to predict the probability of pGGNs developing IACs before surgery. Thus, appropriate treatment and surgery modalities can be selected.

**Table 4:** Details of the predictive model used to calculate the probability of IAC in pGGNs measurements ≤ 2 cm

Risk factors	Estimate	Std. Error	Statistic	OR (95% CI)	*p*
Intercept	-12.759	4.13		0	0.002
Maximum diameter	2.41	0.417	5.784	11.130 (5.044, 25.966)	< 0.001
Age	0.053	0.018	2.871	1.054 (1.018, 1.094)	0.004
Lymphocyte	-0.027	0.308	-0.089	0.973 (0.527, 1.769)	0.929
PNI	0.081	0.042	1.931	1.084 (1.002, 1.183)	0.054
Erythrocyte	0.787	0.543	1.449	2.196 (0.759, 6.471)	0.147
Hemoglobin	-0.025	0.018	-1.414	0.975 (0.941, 1.010)	0.157
SA	0.049	0.024	2.049	1.050 (1.002, 1.100)	0.04
CYFRA21-1	0.209	0.158	1.321	1.232 (0.911, 1.705)	0.186
CEA	0.094	0.112	0.84	1.099 (0.877, 1.366)	0.401
Gender					
Female	Ref.				
Male	0.768	0.47	1.633	2.154 (0.859, 5.458)	0.103
Smoking history					
Non-smoker	Ref.				
Smoker	-0.327	0.435	-0.751	0.721 (0.304, 1.685)	0.453
Blood type					
A	Ref.				
B	0.582	0.368	1.582	1.790 (0.875, 3.722)	0.114
AB	0.857	0.49	1.749	2.356 (0.898, 6.187)	0.08
O	-0.278	0.386	-0.72	0.757 (0.352, 1.609)	0.472
ASA					
1	Ref.				
2	-0.855	0.526	-1.625	0.425 (0.153, 1.222)	0.104
3	0.345	1.586	0.218	1.412 (0.045, 46.131)	0.828
Location					
Centrality	Ref.				
Peripherality	-1.245	0.515	-2.418	0.288 (0.104, 0.794)	0.016
Shape					
Regularity	Ref.				
Irregularity	0.444	0.327	1.358	1.559 (0.819, 2.962)	0.174
Spiculation					
No	Ref.				
Yes	0.253	0.309	0.819	1.288 (0.699, 2.360)	0.413
Vascular penetration sign					
No	Ref.				
Yes	-0.601	0.346	-1.736	0.549 (0.275, 1.074)	0.083
Pleural adhesions					
No	Ref.				
Yes	0.935	0.308	3.034	2.548 (1.398, 4.700)	0.002
Bronchus sign					
No	Ref.				
Yes	0.979	0.373	2.625	2.662 (1.286, 5.576)	0.009
Lobulation					
No	Ref.				
Yes	0.815	0.38	2.147	2.260 (1.078, 4.799)	0.032

*IAC *Invasive adenocarcinoma, *COPD *Chronic obstructive pulmonary diseases, *ASA *American Society of Anesthesiologists, *PNI *Prognostic nutritional index, * NLR *Neutrophil-lymphocyte ratio, *PLR *Platelet-lymphocyte ratio, *MLR *Monocyte-lymphocyte ratio, *dNLR *derived neutrophil-to-lymphocyte ratio, *NLPR *Neutrophil to lymphocyte and platelet ratio, *SIRI *Systemic inflammatory response syndrome, *AISI *Aggregate index of systemic inflammation, *SII *Systemic inflammation index, *PIV *Pan-immune-inflammation value, *LDH *Lactate dehydrogenase, *SA *Serum amyloid, *5'-NT *5'-nucleotidase, *Pro-GRP *Pro-gastrin-releasing peptide, *SCC *Squamous cell carcinoma, *Cyfra21-1 *Cytokeratin 19-fragments, *CEA *Carcinoembryonic antigen, *CA125 *Carcinoma antigen 125,  *NSE *Neuron-specific enolase, *BMI *Body mass index, *FEV1 *Forced expiratory volume in one second, *MVV *Maximal voluntary ventilation

As shown in this nomogram, there are a total of 10 axes, and axes 2–8 represent the seven variables in the prediction model. By plotting a line perpendicular to the highest point axis, the estimated score for each risk factor can be calculated and can be further summed to obtain a total score. The total point axis was then used to predict the probability of IAC for pGGNs measuring ≤ 2 cm before surgery.

### Predictive performance and nomogram validation

**Fig. 4:**
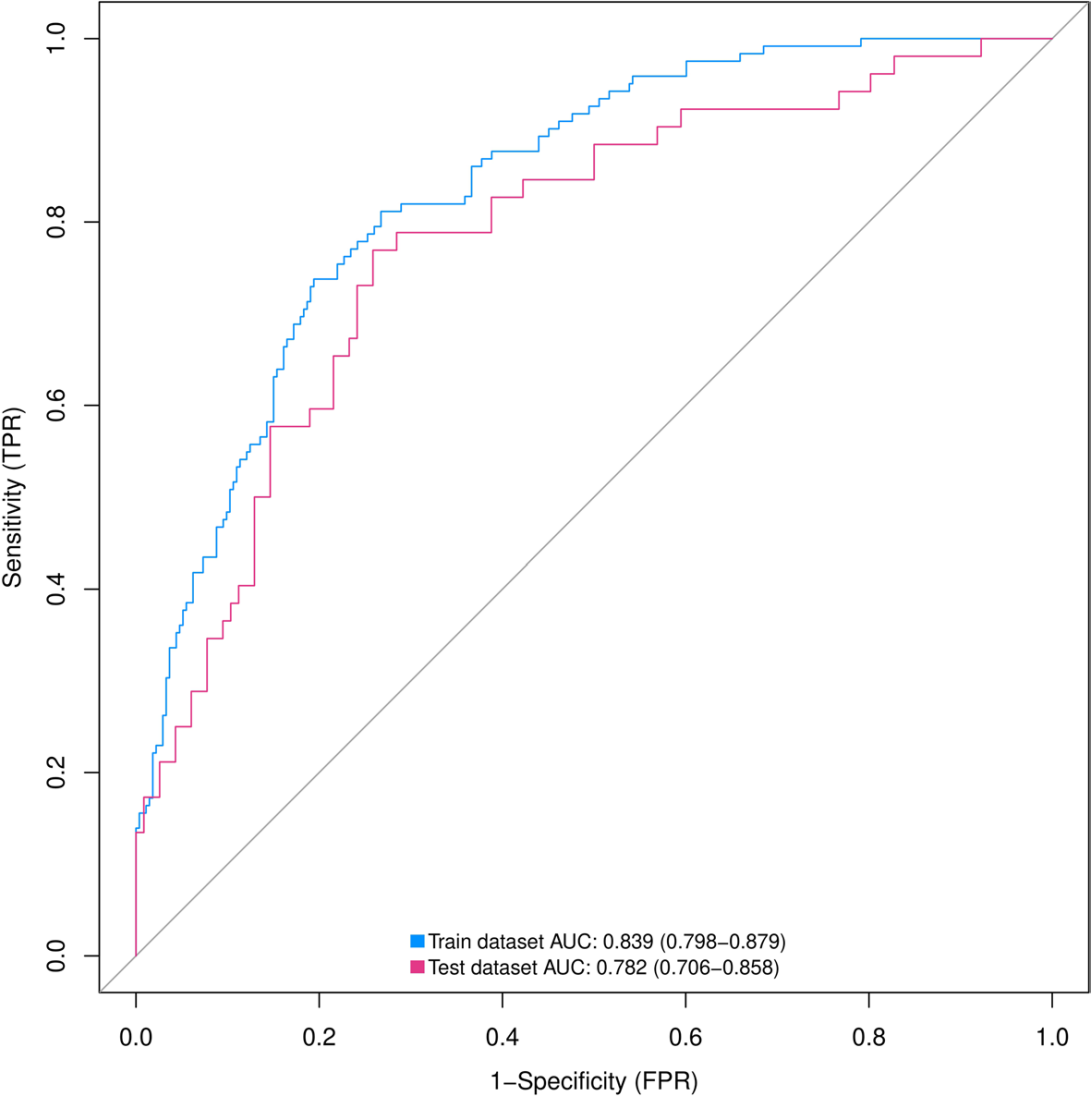
ROC curves of nomograms predicting IAC for pGGN ≤ 2 cm in the training and validation groups. ROC, receiver operating characteristic; AUC, area under the ROC curve; IAC, invasive adenocarcinoma; pGGN, pure ground glass nodule

The discriminatory power of the prediction model and nomogram was assessed via ROC curve analyses. As shown in Fig. 4, the area under the ROC curve (AUC) for the training cohort was 0.839 (95% CI: 0.798–0.879) and for the validation cohort, it was 0.782 (95% CI: 0.706–0.858), indicating that nomogram showed good predictive accuracy. Further, the ROC curve for the training cohort had cutoff, sensitivity, and specificity values of 0.274, 0.811, and 0.733, respectively, indicating excellent performance (Table 5).

**Table 5:** Results of ROC curve for training cohort

Characteristics	Value
Threshold	0.274
Specificity	0.733
Sensitivity	0.811
Accuracy	0.757
TN	200
TP	99
FN	23
FP	73
NPR	0.897
PPV	0.576
FDR	0.424
FPR	0.267
TPR	0.811
TNR	0.733
FNR	0.189
1-specificity	0.267
1-sensitivity	0.189
1-accuracy	0.243
1-NPV	0.103
1-PPV	0.424
Precision	0.576
Recall	0.811
Youden index	1.544
Closest.topleft	0.107

*TP *True positive, *FP *False positive, *TN *True negative, *FN *False negative, *TPR *True positive rate, *FPR *False positive rate, *TNR *True negative rate, *FNR *False negative rate, *PPV *Positive predict value, *NPR *Negative predict value, *FDR *False discovery rate

**Fig. 5:**
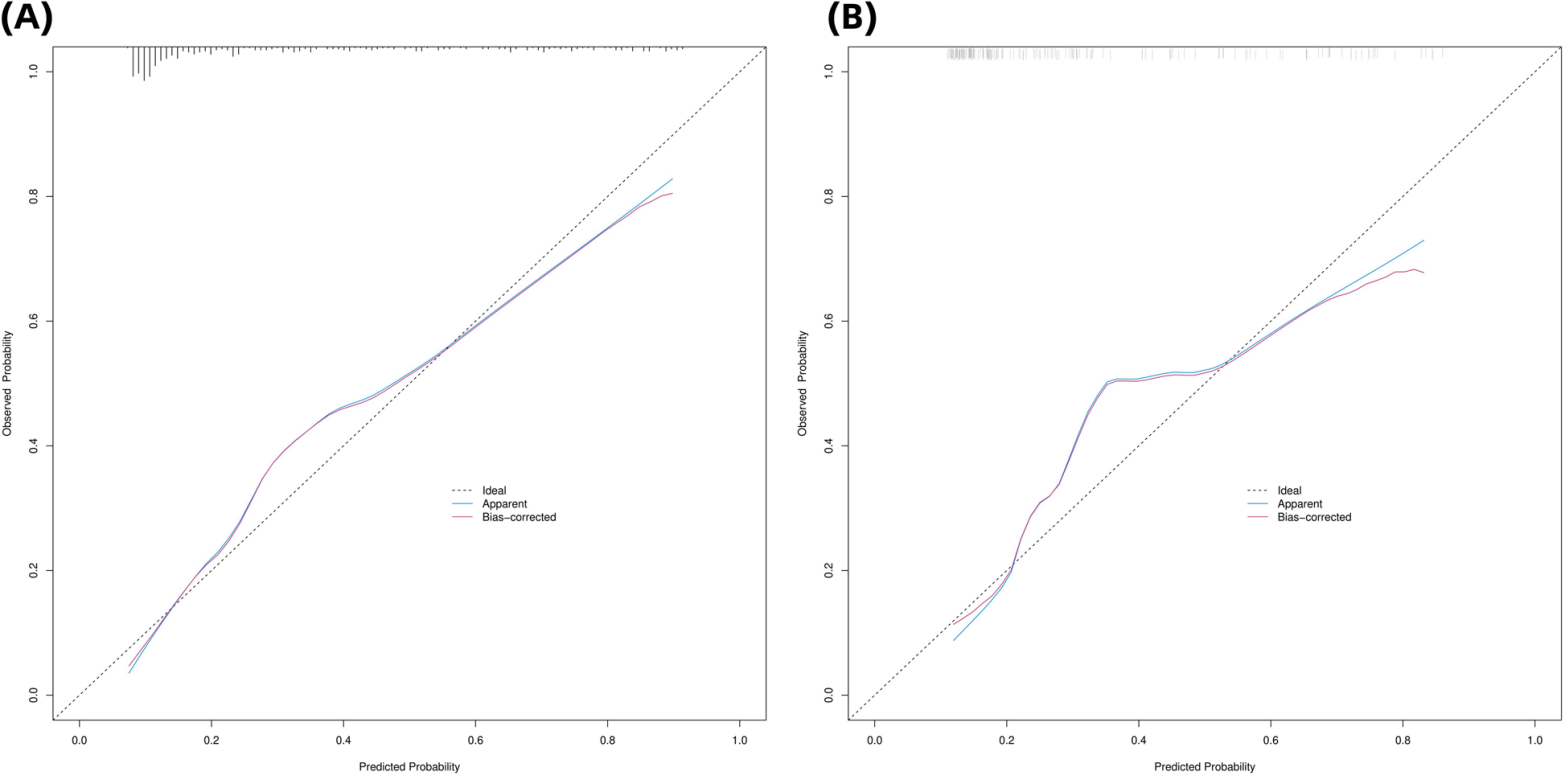
(**A**, **B**): Calibration curves of the prediction nomogram in the training cohort (**A**) and validation cohort (**B**). IAC, invasive adenocarcinoma; pGGN, pure ground glass nodule

We used the Hosmer-Lemeshow test and calibration charts to assess the calibration capability of our model. Thus, the observed Hosmer-Lemeshow test *P*-values for the training and validation cohorts were 0.1071 and 0.2595, respectively, suggesting that the difference between the predicted and actual observed probabilities was not significant. Therefore, the nomogram showed good calibration as indicated by the calibration plots corresponding to the training (Fig. 5a) and validation cohorts (Fig. 5b). Further, the bias-corrected C-indices for the training and validation cohorts were 0.840 and 0.785, respectively.

The X-axis represents the probability predicted by the nomogram and the Y-axis represents the actual probability of pGGN being an ICA within 2 cm. The black dashed line represents the ideal curve, the blue solid line represents the apparent curve (uncorrected), and the red solid line represents the deviation curve corrected by bootstrap method (B = 1000 times).

### Clinical utility of the predictive nomogram

**Fig. 6:**
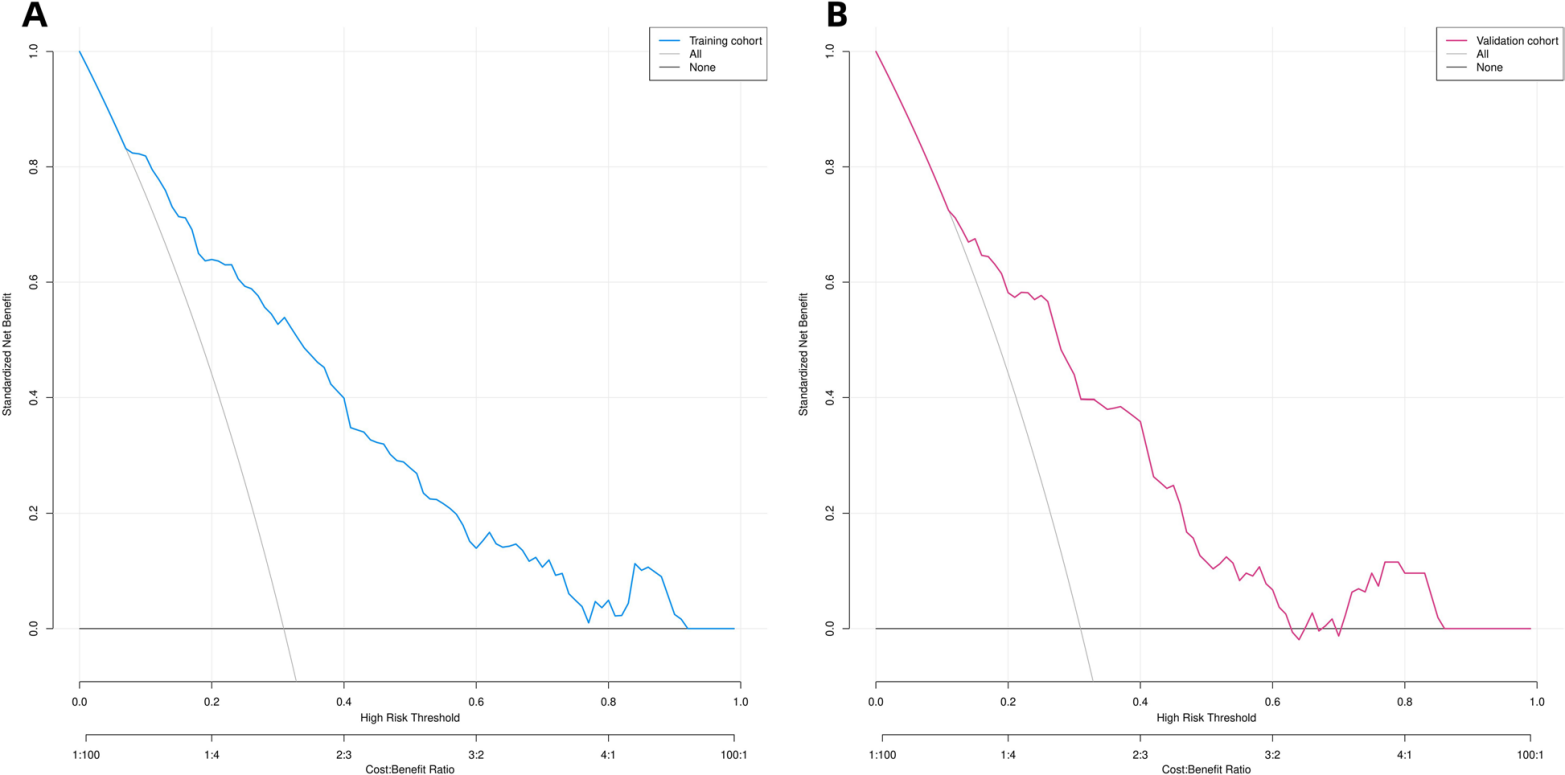
**(A, B)**: Decision curve analysis of predicted nomogram in the training cohort (**A**) and validation cohort (**B**)

**Fig. 7:**
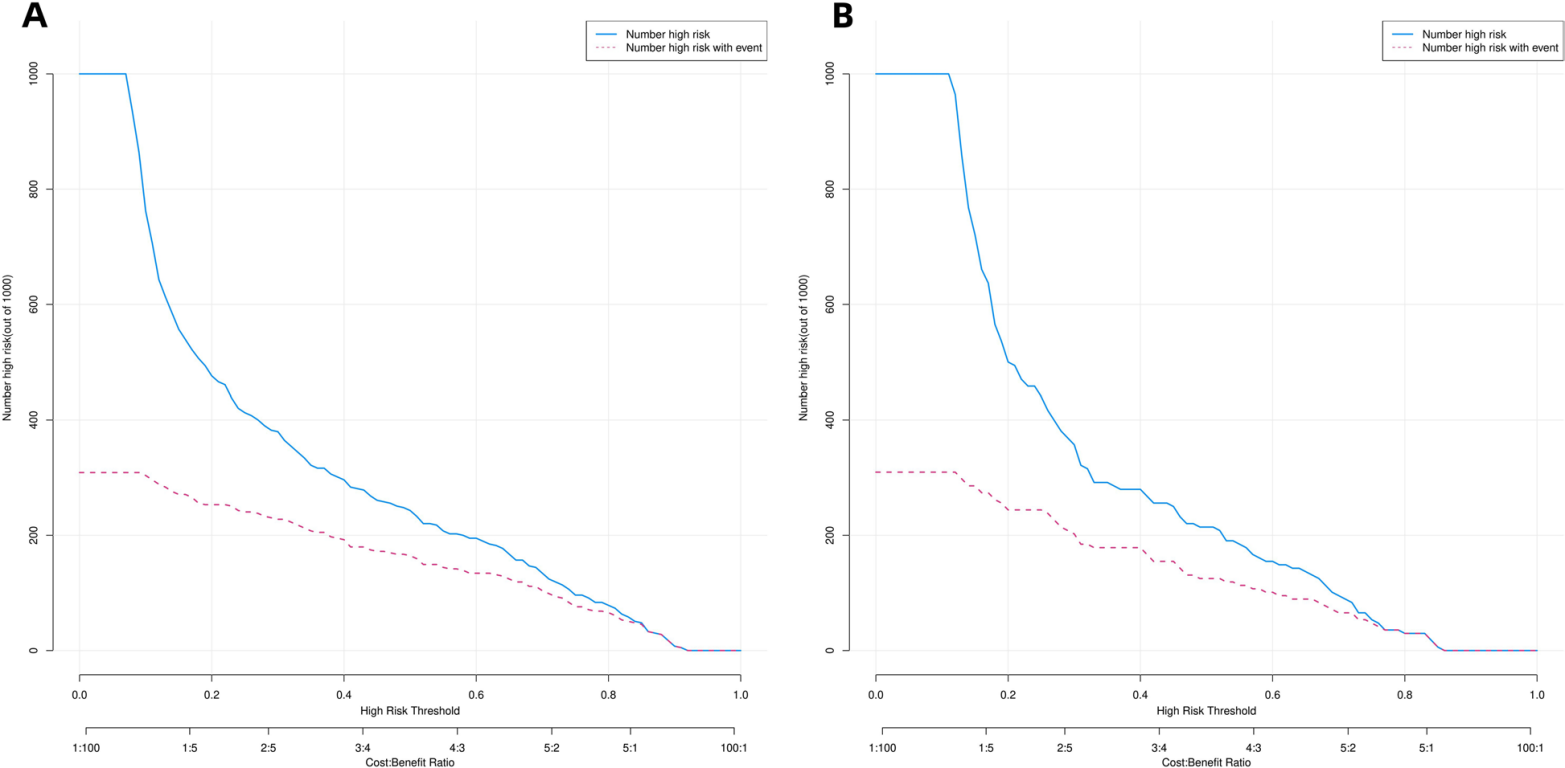
(**A**,** B**): Clinical impact curves of predicted nomogram in the training cohort (**A**) and validation cohort (**B**)

We performed DCA to assess the clinical utility of the predictive nomograms. As shown in Fig. 6a and b, the nomogram provided a greater net benefit and wider threshold probabilities for predicting the risk of IACs in pGGNs measuring ***≤*** 2 cm in both the training and validation cohorts, demonstrating its clinical utility. Additionally, our clinical impact curve (Fig. 7) showed that a high benefit ratio could be obtained within a probability threshold of 0.5–1.0. This observation suggested that the present model can be used in clinical practice to predict the probability of IACs developing in small pGGNs, helping surgeons make better clinical decisions.

The y-axis measures the net gain, and the black line represents the hypothesis that pGGNs within 2 cm are non-IACs in nature, and the gray line represents the hypothesis that pGGNs measuring ≤ 2 cm are IACs. The blue line in Fig. 7A represents the training cohort, and the red line in Fig. 7B represents the validation cohort.

The horizontal coordinate is the probability threshold and the vertical coordinate is the number of people. The blue line indicates the number of people whose pGGNs were judged by the model to be IAC at different probability thresholds; the red line indicates the number of people whose pGGNs were judged by the model to be IAC and nodal true IAC at different probability thresholds. At the bottom, the cost: benefit ratio is also added, indicating the ratio of loss to benefit at different probability thresholds.

## Discussion

The definitive pathological diagnosis of pGGN with diameter ***≤*** 2 cm is relatively challenging owing technical limitations and the potential risk of complications when performing coarse needle aspiration biopsy for pGGN [34, 35]. With the increasing number of cases of early stage lung cancer presenting as pulmonary pGGNs, it is crucial to determine whether a pulmonary pGGN is an IAC; such information is vital for selecting relevant treatment options for patients. In this study, we showed that the percentages of IAC in pGGN were 30.9%, 30.9%, and 31.0% for the total, training, and validation cohorts, respectively. Attempts have been made in previous studies to distinguish IAC from pre-infiltrative lesions; however, these studies did not include benign GGN [18–26]. Further, studies have also been conducted to analyze the differentiation of solid, partial, and GGN nodules [27, 28]. However, clearly distinguishing IAC from non-IAC in pGGN remains challenging. Therefore, in this study, we aimed to explore the potential predictors that can be used to distinguish IAC and non-IAC in pGGN. Thus, we identified seven correlated factors, namely, maximum tumor diameter, age, SA, pleural effusion sign, bronchial signs, tumor location, and lobulation.

Nodule size is an important parameter for assessing GGN invasiveness. Several investigations have shown that increasing adenocarcinoma aggressiveness is associated increasing pGGN lesion size increases [21, 36]. It has also been reported that a critical nodule size of 1 cm is optimal for predicting aggressive pGGN with 100% specificity [20]. However, Wu et al. demonstrated that nodule size cannot be used to distinguish between infiltrative and pre-infiltrative lesions; the mean nodule size in their study was < 1 cm [21]. In our predictive nomogram, the maximum tumor diameter was identified as the most significant risk factor, consistent with the results of previous studies [37, 38]. Notably, in China, the prevalence of IAC in pGGN is higher than that in pGGN of a similar size in Western countries (24%) [4]. These observation suggests that, owing to the high risk associated with IAC, biopsy or surgery should be considered as soon as possible when pGGN size is > 1 cm.

Hu et al. indicated that age ≥ 60 years is a risk factor for IAC [39]. Consistent with their findings, the results of this study suggested that the probability of IAC in pGGN increases with age. However, this risk factor is not well recognized. Therefore, clinicians should be cautious when pGGN is observed in older patients. Further, such patients should undergo more frequent follow-up CT scans and be considered for biopsy or surgery.

Previous studies have also demonstrated that the CT features of pulmonary nodules can be used to assess their aggressiveness. These imaging features included lobulation, spiculation, bronchus signs, cavitation signs, pleural adhesion signs, and nodule shape [24, 25, 27, 40–52]. Furuya et al. reported that 82% of lobulated nodules and 97% of acinar nodules are malignant [53]. Lobulation and spiculation of pGGN are also more common in invasive lesions than in pre-infiltrative lesions [18]. Further, Lee et al. found that lobulation is more common in IAC than in pre-infiltrative lesions [42]. However, lobulation was not included as a risk factor for malignant lung nodules in the Herder model [51]. In this study, we identified lobulation as a risk factor for the occurrence of IAC in pGGN; however, no significant differences were observed in this regard with respect to spiculation. A possible reason for this observation is the limited number of nodules with spiculations included in the study. The bronchus signs observed in this study constituted another CT feature that showed association with malignancy. Bronchial signs were more frequently observed in patients with malignant GGN than in those with benign GGN. Reportedly, patients with IAC present with bronchial signs more frequently than those with AIS [45, 54]. Thus, based on our results, bronchial signs were identified as significant predictors of IAC (*P* = 0.009).

Pleural effusions have rarely been associated with the aggressiveness of pGGN in the literature. Our study demonstrated that pleural effusion signs on CT images can be used as a predictor of IAC in pGGN. At initial diagnosis, approximately 15% of patients with lung cancer present with pleural effusion, while 50% of patients at the advanced stages of the disease present with pleural effusion [55, 56]. If the pleural effusion is malignant, the patient’s prognosis is poor. Therefore, if signs of pleural effusion are observed in CT images for patients with pGGN, treatment should be considered as soon as possible.

The distinction between the centrality and peripherality of nodules is also a significant indicator of the risk of IAC in pGGN. Our results suggested that central pGGNs are more likely to be aggressive than peripheral pGGNs. However, previous studies with a focus on the relationship between centrality and nodal aggressiveness did not show any significant association in this regard [57]. Therefore, a multicenter clinical study with a large sample size is required to validate our findings.

Amylase production in lung cancer has been identified via pathological or immunohistochemical analyses, and it has also been confirmed that its serum level decreases after resection. Previous pathological and biochemical studies also support the existence of a mechanism by which lung cancer tissues produce amylase [58, 59]. Further, several case reports have described high serum amylase levels in patients with lung cancer [60–70]. However, this study is the first identify SA as a risk factor for predicting the probability of IAC occurrence in pGGN. In an immunohistochemical study, amylase levels in lung cancer tissues were found to be higher than those in normal lung tissues [63]. In contrast, other studies have shown that inflammatory as well as normal lung tissue can also produce amylase [58, 71] and that increased positive staining for amylase in lung cancer tissue is not associated with hyperamylasemia [72]. Adenocarcinoma is the predominant histological type of amylase-producing lung cancer [59, 73]. Our results revealed a positive correlation between SA concentration in vivo and nodule invasiveness, i.e., a higher SA concentration resulted in a higher probability of IAC occurrence in pGGN. Therefore, SA may be a new indicator for monitoring and evaluating patient prognosis.

Additionally, this study showed that multiple serum tumor biomarkers (CEA, CYFRA21-1, SCC, NSE, and CA125) had no significant value in distinguishing IAC from pGGN. The reason for their ineffectiveness may be that in early-stage lung adenocarcinomas, tumor proteins are rarely secreted into the bloodstream.

The performance of our constructed predictive model was found to be comparable with those of previously published predictive models by a considerable margin. First, we introduced benign tumors and combined them in a non-IAC cohort. This grouping method showed great value for guiding clinical decision making given that pGGNs observed in clinical practice cannot be completely excluded as benign. Second, we randomly divided the collected cases into the training and internal validation cohorts, thereby strengthening our conclusions. Third, we collected comprehensive clinical and imaging data and provided a clear pathological diagnosis for each patient. Fourth, the corrected C-index value for the training cohort of the model was 0.837, and the Hosmer-Lemeshow test *P*-value was 0.1071, indicating good performance. Further, the ROC, calibration, and DCA curves performed well, and the accuracy and reliability of the model were satisfactory. Fifth, all the important risk factors in our nomogram are readily available and prevalent in clinical practice.

This study had some limitations. First, this was a single-center retrospective study that included only surgically resected pGGNs; therefore, validation bias was inherent in our study design. Second, this was a retrospective cross-sectional study. Until present, no longitudinal study assessing nodal growth has been reported. Thus, further studies are required in this regard. Third, the definition of pGGN and the criteria for pathological diagnosis may vary among physicians. Fourth, the subjectivity of radiologists may have led to different judgments regarding the characteristics of the examined pulmonary nodules. Fifth, only internal validation was conducted. Therefore, further studies with the recruitment of more patients for external validation are necessary. Additionally, the applicability of our predictive nomogram remains to be confirmed in multicenter prospective clinical trials with large sample sizes.

## Conclusion

In this study, we showed that the maximum tumor diameter, age, SA, pleural effusion sign, bronchial sign, tumor location, and lobulation were predictors of IAC in patients with pGGN in the lungs. We also developed and validated a novel, easy-to-use nomogram for predicting the risk of IAC occurrence in patients with pGGN measuring ***≤*** 2 cm in diameter, based on certain influencing factors. This tool may be used to guide clinicians in developing specific and individualized treatment strategies for patients.

### Supplementary Information

**Supplementary material 1.**

## Data Availability

The data that support the findings of this study are available on request from the corresponding author.
